# In Vitro Mycorrhization for Plant Propagation and Enhanced Resilience to Environmental Stress: A Review

**DOI:** 10.3390/plants14142097

**Published:** 2025-07-08

**Authors:** Hassna Radi, Meriyem Koufan, Ilham Belkoura, Tayeb Koussa, Mouaad Amine Mazri

**Affiliations:** 1 Agro-Biotechnology Research Unit, Regional Center of Agricultural Research of Marrakech, National Institute of Agricultural Research, Avenue Ennasr, BP 415 Rabat Principale, Rabat 10090, Morocco; hassna98radi@gmail.com; 2 Laboratory of Plant Biotechnology, Ecology and Ecosystem Valorisation, CNRST-URL10, Faculty of Sciences, University Chouaïb Doukkali, El Jadida 24000, Morocco; koussa.t@ucd.ac.ma; 3 Natural Resources and Local Products Research Unit, Regional Center of Agricultural Research of Agadir, National Institute of Agricultural Research, Avenue Ennasr, BP 415 Rabat Principale, Rabat 10090, Morocco; meriyem.koufan@inra.ma; 4 In Vitro Culture Laboratory, Department of Basic Sciences, National School of Agriculture, BP S/40, Meknes 50001, Morocco; bilham@enameknes.ac.ma

**Keywords:** abiotic stress, biotic stress, micropropagation, in vitro mycorrhization

## Abstract

Arbuscular mycorrhizal fungi (AMF) play a key role in enhancing plant stress tolerance, nutrient uptake, and overall health, making them essential for sustainable agriculture. Their multifaceted contributions to the rhizosphere—through biofertilization, bioprotection, and biostimulation—have led to growing interest in their application. In recent years, in vitro mycorrhization has emerged as a promising approach for the rapid propagation of economically and ecologically important plant species, offering improved agronomic and physiological traits as well as increased resilience to environmental stressors. However, challenges remain in achieving consistent AMF-plant symbiosis under in vitro conditions across diverse species. This review highlights the potential of in vitro mycorrhization as a controlled system for investigating AMF interactions and their impact on plant development. Various in vitro mycorrhization systems are described and discussed, along with their applications in the mass production of AMF propagules and mycorrhizal plants, and their role in enhancing the acclimatization of micropropagated plantlets to ex vitro conditions. The role of in vitro mycorrhization as an effective tissue culture approach that integrates plant propagation with enhanced resilience to environmental stress is emphasized. The factors influencing the success of in vitro mycorrhization and strategies for the large-scale production of AMF propagules and mycorrhizal plants are explored. Although research in this area is still limited, existing studies underscore the potential of in vitro mycorrhization to enhance plant tolerance to abiotic and biotic stresses—an increasingly urgent goal in the context of climate change and global food security.

## 1. Introduction

In recent years, mycorrhizal fungi have gained considerable attention as key soil microorganisms in modern agriculture due to their indispensable role in sustainable agriculture. They are increasingly recognized as biofertilizers and biological control agents, offering a sustainable alternative to chemical fertilizers and synthetic pesticides [1,2]. Mycorrhizal fungi enhance nutrient uptake, stimulate photosynthesis, promote hormone production, protect against soil-borne pathogens, and improve drought resistance and soil structure [3]. This is achieved through the formation of extensive hyphal networks and modification of root architecture, which together increase the root surface area and enable plants to access water and nutrients more efficiently [4]. Additionally, mycorrhizal fungi enhance hormonal signaling, leading to increased chlorophyll production and improved carbon assimilation [5,6]. They also protect the host plant by competing for space and resources with soil-borne pathogens, producing antimicrobial compounds, regulating pathogenesis-related genes, and inducing systemic resistance [7,8]. Furthermore, mycorrhizal fungi secrete glomalin, a glycoprotein that promotes soil particle aggregation, thereby improving soil structure and stability [9].

Mycorrhiza refers to the obligate symbiotic association between fungi and the roots of approximately 90% of vascular plant species [10]. This ‘obligatory’ natural and mutualistic association occurs because the fungi cannot produce their own organic matter and rely entirely on the host plant for organic carbon, primarily in the form of photosynthetic carbohydrates. In return, fungi support the plant by enhancing its nutrient and water uptake, which boosts plant growth and improves resilience to abiotic and biotic stresses [2].

Substantial advances have recently been made in the study of mycorrhizal phylogeny and taxonomy. Many studies have highlighted the remarkable diversity of mycorrhizal associations, as most vascular plants are colonized by mycorrhizal fungi [11,12]. Traditionally, mycorrhizas have been classified based on the morphological characteristics of the fungal structures and their associations with host plants. This classification divides mycorrhizas into two major types: ectomycorrhizas and endomycorrhizas. Some research has subdivided mycorrhizas into several categories, including arbutoid, ericoid, orchid, monotropoid, and arbuscular mycorrhizae [13].

Endomycorrhizae, which include arbuscular mycorrhizal fungi (AMF) as one of their main groups, belong to the *Glomeromycota* phylum. These fungi are widely distributed and are the most prominent mycorrhizal type, forming associations with approximately 80% of vascular plants [14]. The hyphae of AMF differentiate into highly branched structures known as arbuscules, which develop inside plant root cells [15]. These arbuscules are the sites of bidirectional exchange between the fungi and the host plant [16]. AMF are mainly categorized into two types based on their hyphal structure: *Arum* and *Paris* types. In the *Arum* type, the fungi form linear hyphae that grow through the intercellular spaces and penetrate cortical cells with short lateral branches to produce arbuscules. The *Paris* type is characterized by coiled, intracellular hyphae that spread directly from cell to cell within the cortex, forming large coils with small intercalated arbuscules [17,18]. Other types include the Hepatic series, which resembles *Paris*-type associations but features arbusculate structures that are not arranged in distinct layers, and the Orchid series, characterized by the formation of tightly coiled intracellular hyphae known as ‘pelotons’ [17].

The establishment of the arbuscular mycorrhizal (AM) symbiosis goes through different stages, which can be divided into two distinct processes: (i) presymbiotic communication and signaling (the asymbiotic stage), where the plant exudes a range of signaling molecules (e.g., strigolactones (SLs), carotenoid-derived molecules) that trigger spore germination, fungal growth, and branching, as well as the fungus’s encounter with the host plant [19]. The presence of the host is critical, as its absence limits hyphal development. In response, Myc factors (e.g., lipochitooligosaccharides, chitooligosaccharides) are secreted by AMF, which aid in host recognition and trigger molecular responses that ensure the establishment of a successful symbiosis [20]. The second process, the (ii) symbiotic stage, begins when the fungal hyphae make contact with the plant root and differentiate into an appressorium, which attaches to the root epidermis. The hyphae then penetrate the root cortex via a tube-like structure called the pre-penetration apparatus, which is formed by invaginations of the plant cytoplasm. Once inside, the fungus grows both inter- and intracellularly, forming arbuscules or hyphal coils within the cortex [21]. Finally, AMF develop an external mycelium that extends out of the root, exploring the soil for mineral nutrients. This external mycelium can also colonize other susceptible roots by producing spores, thus completing the fungal life cycle [22].

Plant propagation by tissue culture, also known as in vitro propagation or plant micropropagation, is an important aspect of plant biotechnology. It involves the process of regenerating a complete plant from a single cell, tissue, or organ cultured under appropriate culture conditions. This is made possible by the concept of plant cell totipotency, which refers to the ability of a single cell to develop and regenerate into a fully functional organism [23]. Several culture systems are used in plant micropropagation, with the most common being somatic embryogenesis, organogenesis, and microcuttings. Somatic embryogenesis is the process by which bipolar structures, known as somatic embryos, are formed from cells without gametic fusion. These embryos can then develop into complete plants. The success of this process is influenced by various factors and their interactions [24,25]. Adventitious organogenesis involves the induction of adventitious shoot buds from an explant, which are then cultured under optimal conditions to promote growth. These buds are elongated, rooted, and eventually develop into complete plants [26,27]. Propagation by microcuttings refers to the process by which uni- or binodal segments taken from stem cuttings are cultured in vitro to promote axillary shoot development. Multiplication is achieved by sequential segmentation of elongated shoots, followed by root induction and plantlet acclimatization [28].

Plant micropropagation is a widely used technology in agriculture and research, offering numerous applications such as rapid and large-scale propagation, stress resilience enhancement, genetic improvement, synseed production, phytochemical and bioactive compound production, in vitro conservation, and efficient commercial plant production [29,30,31,32,33,34,35,36,37,38]. In recent years, in vitro mycorrhization of plants has emerged as a promising technology that combines plant propagation with mycorrhizal inoculation under in vitro conditions. Successful in vitro mycorrhization has been reported for various species, including Banana [39], potato [40], pear [41], date palm [42], argan [43], and bamboo [44]. This approach holds great potential for the rapid and large-scale propagation of plants with enhanced resilience to abiotic and biotic stresses.

This paper provides an overview of recent advances in the in vitro mycorrhization of plants. It outlines the various methods used for spore extraction, culture, production, and establishment of in vitro associations with host plants, discusses the role of in vitro mycorrhization in enhancing resistance to abiotic and biotic stresses, and summarizes key findings related to the successful mycorrhization of economically important plant species.

## 2. Mycorrhizae in Agrosystems

AMF are among the most widespread soil microorganisms and play a crucial role in sustainable agriculture. Their multiple beneficial effects in the rhizosphere contribute to biofertilization, bioprotection, and biostimulation. As biofertilizers, AMF improve nutrient acquisition by extending the effective root surface area through their hyphal networks. They enhance the availability and uptake of poorly mobile ions, such as phosphate, by facilitating their transport to plant roots [45]. This not only reduces the need for chemical fertilizers but also supports better plant nutrition under nutrient-poor or degraded soils. Through bioprotection, AMF enhance plant tolerance to a range of abiotic stresses such as drought, salinity, heavy metal toxicity, and temperature extremes. They achieve this by regulating ion homeostasis, boosting antioxidant defenses, and modulating stress-related gene expression [46,47,48]. Furthermore, AMF act as biostimulants by influencing root architecture, enhancing photosynthetic efficiency, and altering hormone levels such as abscisic acid (ABA), which helps in managing stress responses [49]. The synergistic effects of AMF colonization not only improve plant health and productivity but also contribute to soil structure and microbial biodiversity, making them essential agents in the development of resilient and sustainable agroecosystems [50].

### 2.1. Enhancing Nutrient Uptake and Promoting Plant Growth

Mineral deficiencies, along with water scarcity and disturbances to soil biofunctions, are major constraints on agroecosystem productivity. While fertilizer application can boost yields, its continuous use leads to significant environmental concerns [51]. Numerous studies have demonstrated the positive effects of AMF on various plant species, including grapevine [52], argan [53], tomato [54], and banana [55], showing enhanced vegetative growth compared to non-inoculated plants. AMF contribute to improved growth and yield by increasing nutrient uptake [56]. They achieve this by facilitating the transfer of soil-bound nutrients to the plant through root cortical arbuscules, which serve as sites of nutrient exchange [22].

In nutrient-deficient plants, especially those lacking in nitrogen, root exudation stimulates AMF colonization and symbiosis [57]. The same mechanism applies to phosphorus-deficient plants, which, although abundant in soil, is poorly soluble and difficult for plants to absorb [57,58,59]. AMF can increase phosphorus solubility by secreting enzymes (e.g., phosphatases, lyases) and organic acids (e.g., gluconic, citric acids). Mycelial secretions of AMF have been found to contain organic acids, carbohydrates, amino acids, plant hormones, and various other compounds [60]. In addition to phosphorus, AMF also acquire and provide other essential nutrients to plants, including potassium (K), copper (Cu), iron (Fe), and zinc (Zn) [61], which makes AMF effective biofertilizers.

On the other hand, AMF can influence the production of secondary metabolites, such as carotenoids and flavonoids, enhancing the nutritional quality of crops and promoting healthier food production [62]. AMF also promote disease resistance and plant growth by secreting plant growth hormones (e.g., auxins, cytokinins, and gibberellins) and regulating hormone levels [60].

Additionally, AMF improve water absorption in plants due to the fine network of hyphae, which extends into small pores in the soil, enhancing the surface area for water uptake [63]. It was reported that AMF-facilitated water uptake increases photosynthesis, transpiration rates, leaf area, and relative water content (RWC) in tomato plants [64]. Thus, AMF play a critical role in supporting sustainable agriculture, particularly by enhancing plant growth and improving resilience to environmental stress.

Recent advances in multi-omics approaches have contributed to elucidating the complex molecular networks underpinning AMF-mediated plant responses to environmental stress and nutrient uptake, offering valuable insights for sustainable agriculture. Transcriptomic and metabolomic analyses demonstrated that isolates of *Rhizophagus intraradices* and *Funneliformis mosseae* induced distinct molecular responses in *Pyrus betulaefolia* roots, modulating gene expression related to root metabolism and altering pathways involved in carbohydrate metabolism, fatty acid and amino acid biosynthesis, and flavonoid production [65]. Similarly, AMF inoculation in *Populus cathayana* was shown to regulate the expression of nitrogen transporter genes and enhance the activities of glutamine synthetase and glutamate synthetase. Under drought stress, AMF improved plant growth, gas exchange parameters, antioxidant enzyme activities, total nitrogen content, and the levels of both water-soluble and membrane-bound proteins, underscoring its critical role in nitrogen uptake and utilization [66]. In maize, integrated physiological, transcriptomic, and lipidomic analyses revealed that AMF colonization under low-temperature stress resulted in substantial remodeling of lipid classes and differential expression of 702 genes, contributing to enhanced membrane stability and cold tolerance [67]. Metabolomic profiling of *Juglans regia* under drought conditions revealed that AMF symbiosis differentially regulated over 150 metabolites, including key components of the phenylalanine metabolism and oxidative phosphorylation pathways [68]. Additionally, studies in *Malus domestica* and *Elymus nutans* revealed that AMF associations promote host resilience to abiotic stress through the coordinated upregulation of genes and metabolites involved in sugar metabolism, fatty acid biosynthesis, and flavonoid pathways [69,70].

### 2.2. Improving Soil Structure

Soil degradation can result from various processes, including soil erosion, compaction, loss of biological activity, and acidification, all of which negatively impact soil quality. Soil structure is a critical property that ensures the retention and transport of water, gases, and nutrients. This is vital not only for maintaining soil porosity and resistance to erosion but also for preserving soil productivity [71]. Furthermore, sustainably managed soils play a significant role in mitigating climate change through carbon sequestration, promoting vegetation establishment, and supporting soil restoration. In contrast, soil degradation is often exacerbated by the loss of soil organic matter, which disrupts the formation and stabilization of soil aggregates, reducing the soil’s capacity to capture carbon from the atmosphere [72].

Soil aggregates, the primary structural units of soil, are crucial for maintaining soil stability. When their stability is compromised, it leads to degradation of the soil structure, hindering water and nutrient cycling [73]. The critical role of AMF in facilitating soil aggregation and supporting sustainable agricultural ecosystems was previously reported [74]. AMF contribute to soil aggregation and stability both physically, through their extensive mycelial networks that entangle and interconnect soil particles, and chemically, by producing and secreting glomalin [22,72,75]. Glomalin and other glomalin-related soil proteins (GRSPs) are fundamental to soil quality and act as stable carbon sinks. They persist longer in the soil than plant-derived carbon, contribute up to 20 times more to soil organic carbon than microbial biomass carbon, and enhance carbon sequestration [76]. Moreover, due to their recalcitrance, GRSPs play a key role in heavy metal sequestration, an essential process for the ecological restoration of soils degraded by mining and smelting activities [75]. GRSPs help stabilize and reduce the availability of toxic metals by converting, accumulating, and transferring them, thus aiding in soil remediation [77].

### 2.3. Enhancing Resilience to Abiotic and Biotic Stress

In recent years, abiotic and biotic stresses have caused significant yield losses in many cultivated crops. To address these challenges, mycorrhizal inoculation was suggested as a potent strategy to mitigate stress constraints in economically important plant species [2]. AMF can enhance plant resistance to pathogens through various mechanisms, including competition with soil-borne pathogens for nutrients and photosynthates, altering rhizosphere interactions to reduce pathogen populations, and activating plant defense systems [78]. Moreover, some studies have demonstrated AMF potential as biocontrol agents against nematodes in economically important crops [16].

The effects of AMF on disease symptoms have been studied for a variety of pathogens, including fungal species such as *Fusarium oxysporum*, *Verticillium dahliae*, *Erwinia carotovora*, and *Ralstonia solanacearum* [79], as well as bacterial pathogens such as *Xanthomonas translucens* and *Candidatus Liberibacter solanacearum*, and viruses including *Tomato Yellow Leaf Curl Sardinia Virus* (TYLCSV) and *Cucumber Mosaic Virus* (CMV) [80,81]. The bioprotective potential of AMF appears to be highly dependent on several factors, including the type and lifestyle of the pathogen, as well as the genotypes of both the pathogen and the host plant [82,83].

In addition to their role in bioprotection, AMF have also been shown to enhance plant tolerance to abiotic stresses such as salinity, drought, extreme temperatures, and heavy metals. Some studies have found that mycorrhizal plants can modulate the biosynthesis of phytohormones, regulate the accumulation of osmoprotectants (e.g., proline) to facilitate osmotic adjustment, and upregulate both primary and secondary metabolites [84]. AMF also enhance the activity of antioxidant systems, which contribute to improved stress resilience [63]. Additionally, AMF help plants tolerate heavy metal toxicity by sequestering metals through compartmentalization and immobilization in hyphal cells, as well as by metal chelation, creating a more favorable environment for plant growth and development [85]. Due to their extensive hyphal network, AMF increase the absorption surface of plant roots, thus improving water and nutrient uptake, which enhances plant tolerance to drought stress [2].

## 3. In Vitro Mycorrhization: System Description and Evaluation of Plant–Fungus Interactions

Since AMF have become increasingly important for sustainable agriculture, understanding the mechanisms behind plant–fungi interactions has become a critical priority. This requires the development of advanced techniques for studying these associations. In vitro culture is one of the most promising approaches used by researchers to explore various aspects of mycorrhizal symbiosis. This method enables non-destructive, long-term observations, providing more detailed insights into the biology of AMF. Additionally, it allows for the large-scale production of mycorrhizal fungi and plants under controlled conditions in a relatively short time [2,86,87].

Although many of the protocols and culture media referenced in this section were developed several decades ago, they continue to be standard tools in current in vitro mycorrhization research due to their proven effectiveness and broad applicability.

### 3.1. Isolation Methods and Culture Conditions

The isolation of AMF is a crucial first step for the successful culture and mass production of mycorrhizae. They can be isolated either from field soil or trap cultures. Several methods have been developed for isolating AMF spores, which have been compared [88]. These methods include the immersion tube method [89], the direct inoculation method [90], the soil plate method [88], the dilution plate method [91], and a modification of the Rossi-Cholodny buried slide technique [88]. These methods were later compared to the soil washing method, which allowed for a more accurate assessment of active fungi in rhizosphere soil [92]. Agar media such as potato dextrose agar (PDA), Czapek, or yeast extract agar are used in these isolation methods.

Other methods have been developed and compared for mycorrhizal extraction, including flotation-adhesion [93], airstream fractionation [94], and differential water/sucrose centrifugation [95], among others. However, the wet sieving and decanting method remains the most effective and widely used. Originally employed for extracting plant nematodes, this technique was later adapted to extract *Endogone* spores from soil [96]. The method typically involves suspending the soil sample in water, gently stirring to release AMF spores, and allowing heavier particles to settle. The suspension is then poured through a series of sieves with different mesh sizes. The resulting liquid is collected in a tube and centrifuged.

In a modified protocol, the obtained supernatant may be passed through the smallest sieve, and the sieve content is mixed with a sucrose solution. The mixture is then subjected to a second centrifugation. In other variations, after the first centrifugation, the supernatant is discarded and replaced with a sucrose solution, followed by another centrifugation. After the second centrifugation, the supernatant is transferred to a 40 μm sieve and rinsed with water to remove excess sucrose. The spore suspension is then transferred to a Petri dish and examined under a stereomicroscope to isolate the spores using a flattened needle [96,97,98].

Spores can be morphologically identified based on their shape and their reaction to polyvinyl alcohol/lactic acid/glycerol (PVLG) or a mixture of PVLG and Melzer’s reagent [99]. These morphological traits are then compared to the descriptive features of known species available in the International Collection of Vesicular Arbuscular Mycorrhizal Fungi (INVAM: https://invam.ku.edu) [100]. The morphological observations should be complemented by molecular analysis for species-level identification. If spores are not used immediately, they should be stored at 4 °C in distilled water, on water agar, or in a 0.1% MgSO_4_·7H_2_O solution solidified with 0.4% gellan gum to maintain their physiological integrity [101].

#### 3.1.1. Selection Criteria for AMF Inoculum

In addition to being cost-effective, the primary factors for selecting an inoculum formulation include its positive impact on target plant survival and growth. Additionally, the inoculum viability must be maintained during storage to ensure the fungus remains effective after inoculation for optimal plant growth [102]. Various AMF propagules can be used, including spores and sporocarps, mycorrhizal root fragments, and isolated vesicles [103,104]. However, each of these propagules has its own colonization potential. For instance, mycorrhizal root fragments generally have a higher inoculum potential than spores [105], as spores may be dead or have low viability if not collected from a trap culture or the root zone of an actively growing, infected plant [103]. Moreover, the presence of intraradical vesicles in roots has been shown to contribute to higher inoculum potential [106].

In general, two types of inocula—sporocarps/spores and mycorrhizal root fragments with vesicles—are most commonly used to initiate in vitro monoxenic cultures. Sporocarps are typically the preferred propagules for starting monoxenic cultures due to their ability to undergo multiple germinations. Spores are typically used for species that produce few or no vesicles (e.g., *Funneliformis caledonium* and *Funneliformis mosseae*), or for species with large spores (diameter > 150 μm) [107]. Root fragments with vesicles are the preferred type for species such as *Rhizophagus intraradices* and *Rhizophagus prolifer*, which produce small spores (diameter < 100 μm) or are characterized by low germination rates and slow hyphal growth [107].

Once isolated, the propagules must be surface sterilized to ensure a contaminant-free inoculum. Several disinfectant solutions are commonly used, such as 95% ethanol and 6% calcium hypochlorite for disinfecting mycorrhizal root segments, with disinfection typically carried out using an ultrasonic apparatus under a laminar flow hood [108]. For spore sterilization, a 2% chloramine T solution combined with Tween 20 is often used, followed by rinsing in an antibiotic solution containing streptomycin and gentamycin. All surface sterilization steps should be performed on ice to maintain spore dormancy [104].

Among the most frequently cultured AMF species in vitro is *Glomus intraradices*, which is also one of the most productive species [109]. The selection of AMF species should align with the intended application. If the inoculant is meant for general use, it should contain at least one generalist species capable of colonizing a wide range of host plant species. Additionally, the inoculant must contain enough viable propagules to ensure effective AM root colonization. High concentrations of viable propagules are important to compensate for the declining germination rates of AMF propagules during prolonged storage. Finally, AMF inoculants must be free of plant pathogens and other harmful contaminants [110].

#### 3.1.2. Host Root Selection for AMF Colonization

Several root organs have been used as hosts for growing AMF in vitro, including tomato (*Lycopersicum esculentum* Mill.), red clover (*Trifolium pratense* L.), strawberry (*Fragaria x Ananassa* Duchesne), onion (*Allium cepa* L.), chicory (*Cichorium intybus* L.), and other mycotrophic plants [41,104]. However, the carrot hairy root line (*Daucus carota* L.) is the most widely used host for in vitro AMF culture. These hairy roots are obtained through the genetic transformation of carrot roots using *Agrobacterium rhizogenes*, which transfers the root-inducing (Ri) plasmid (specifically the T region of the Ri plasmid, responsible for inducing root formation) into the host plant genome [111,112]. Ri-T-DNA-transformed roots exhibit greater intraradical colonization by AMF and support higher extraradical hyphal development compared to non-transformed roots, which is beneficial for fungal production [113]. This transformation results in increased production of auxins and cytokinins, enhanced cell division, the development of the characteristic hairy root phenotype, and the synthesis of opines, which serve as a food source for the colonizing microorganisms [111]. The altered hormonal balance in the transformed roots enables them to grow rapidly and continuously [113].

It is worth noting that the success of mycorrhizal culture establishment depends on the physiological state of host roots. Even when roots are from the same clone and grown under the same conditions, their behavior can vary, significantly affecting mycorrhizal colonization [104]. Other culture parameters, such as subculture frequency, explant selection, and the orientation of Petri dishes during incubation, must be carefully adjusted for better performance [104].

To standardize and optimize the use of hairy roots in propagating AMF in monoxenic cultures, a framework called *RocTest* was developed [112]. This system helps evaluate the performance of hairy roots and identify the best host–AMF combinations to advance the development of in vitro AMF cultures. The source of the host plant is also a critical factor in hairy root induction, as mature and fresher plant material tends to yield better results for initiating hairy root formation [107].

#### 3.1.3. Culture Medium Choice

The selection of an appropriate culture medium is a critical step for the successful in vitro culture of AMF, as it must support both hyphal growth and spore germination. Two of the most commonly used culture media are the modified Strullu–Romand medium (MSR) [114], and the minimal (M) medium [115], which is a modified version of White medium (MW) [116].

The MSR medium, propped by Declerck et al. [114], is based on Strullu–Romand (SR) medium initially developed by Strullu and Romand [117] and later modified by Diop [118]. It is designed to promote the growth of intracellular mycelium and sporulation of fungi under in vitro conditions [104,113]. On the other hand, the M medium is based on WM, which was developed for tomato root-organ cultures and is commonly used to study primary mycorrhiza formation and colonization [104].

Both media are the result of modifications of those typically used for plant micropropagation [113]. They contain essential micronutrients, macronutrients, vitamins, and sucrose, and are adjusted to a pH of 5.5 before autoclaving. They have been shown to be effective for the culture of a variety of AMF species [104,113].

In addition to MSR and M, WM has also been suggested, as it was found to be particularly suitable for root initiation and growth [119]. Murashige and Skoog (MS) medium [120], the most commonly used culture medium in plant micropropagation [27,121], is also sometimes employed, although it has some drawbacks. Specifically, the ammonium ions present in MS medium can negatively affect root growth by causing a rapid drop in pH [122]. Moreover, most AMF spores studied thus far tend to germinate more readily on media with lower nutrient content. MS medium is not specifically designed to meet the needs of mycorrhizal fungi, which often require specific nutrient conditions for optimal growth and sporulation, particularly in terms of mineral balance. As a result, MS medium at full strength may inhibit spore germination, fungal growth, and mycorrhization.

### 3.2. Culture Systems for Mycorrhizal Fungi Production and In Vitro Mycorrhization

Early studies aiming at growing AMF under axenic conditions faced challenges due to the unavailability of in vitro mycorrhiza cultures and the dependency on host plants [123]. Starting from the late 1960s, various approaches have been developed to facilitate the in vitro culture of AMF. One of the earliest breakthroughs was achieved by Mosse [124], who successfully cultured an *Endogone* species using clover (*Trifolium pratense* and *T. parviflorum*) as a host plant. This work was later extended by Mosse and Hepper [125], who achieved in vitro co-culture involving *Endogone mosseae* and excised roots from tomato (*Lycopersicum esculentum*) and red clover (*Trifolium pratense*). Despite the significance of this development that laid the foundation for controlled studies on AMF biology, it garnered limited attention at the time and these early cultures were not free from contamination [126,127]. A significant advancement occurred in the 1980s and early 1990s with the development of the root organ culture system, which used transformed carrot (*Daucus carota* L.) roots generated via *Agrobacterium rhizogenes* Ri T-DNA [115,128], and the introduction of bi-compartmental Petri dishes enabling the spatial separation of extraradical mycelium from colonized root tissues, thus facilitating experimental manipulation [129,130]. These advances significantly enhanced the utility of the monoxenic culture system, particularly for investigating nutrient transport and fungal physiology, and enabled the successful cultivation of various AMF species. In subsequent years, other plant hosts with transformed roots were also employed to culture AMF effectively [126,127]. Since then, various modifications have been made for propagating mycorrhizal fungi in vitro. Today, two main types of in vitro culture systems are used to produce mycorrhizal propagules monoxenically: (1) the root organ culture (ROC) system and (2) the Autotrophic Culture system (ACS) [2,131]. On the other hand, the Mycelium donor plant (MDP) culture system is the most commonly used method for in vitro mycorrhization of agro-economically important plant species [2].

#### 3.2.1. The ROC System

In vitro mycorrhization is a valuable tool for the large-scale production and commercialization of mycorrhizal fungi [2]. The ROC system is primarily employed to study and cultivate AMF under controlled, sterile conditions. It enables the large-scale production of AMF propagules by providing a contaminant-free environment that supports the consistent and scalable generation of fungal spores and hyphae [2]. This system has advanced research on mycelial organization, as well as on sporulation dynamics and spore ontogeny. Furthermore, it holds promise for producing pure, concentrated inocula and contaminant-free fungal tissues, which are crucial for genetic and physiological studies [132].

Many studies have explored the use of root organs as hosts to grow AMF in vitro. To this end, excised roots (or root organ cultures) from various plants, such as red clover (*Trifolium pratense* and *T. parviflorum*) [125], bindweed (*Convolvulus sepium* L.) [128], carrot (*Daucus carota* L.) [115], tomato (*Lycopersicon esculentum*) [133], strawberry (*Fragaria* × *ananassa* Duch.) [134], and *Medicago truncatula* [135] have been co-cultivated with AMF. The carrot hairy root line, established by Bécard and Fortin in 1988, is the most commonly used host for monoxenic cultivation of AMF [113].

The ROC system involves the association of mycorrhiza with hairy root cultures induced by *Agrobacterium rhizogenes*, a gram-negative bacterium that causes hairy root disease in plants [136]. Root transformation by the soil-borne microorganism *Agrobacterium rhizogenes* enables the mass production of roots. Propagation of roots occurs in a Petri dish containing an appropriate culture medium, typically modified WM or MSR medium [137].

Genetically transformed roots exhibit several advantages over normal roots, including higher secondary metabolite production, faster growth rates, and greater genetic stability. These characteristics contribute to the successful propagation of AMF and the generation of large numbers of fungal isolates [138,139]. It was demonstrated that transformed chicory roots infected with *Rhizophagus irregularis* produced new spores within 21–25 days of association, which were capable of completing their lifecycle and forming associations with new hairy roots, with an average of 2000 spores observed per plate after 5 months [140]. Similarly, three phases of sporulation and an average of 9500 spores produced within 5 months were observed when *Glomus vesicolor* was associated with Ri T-DNA transformed carrot roots, along with extensive root colonization and the formation of numerous arbuscules and vesicles [114].

Although this method requires advanced technical expertise and is relatively costly, it enables the production of aeroponically developed, colonized roots that can be sheared, maintained free of extraneous microorganisms, and preserved in vitro [141].

An extension of the ROC system is the bi-compartmental or split system [130]. This system consists of two compartments: the proximal or root compartment (RC) for the association of roots with AMF, and the distal or hyphal compartment (HC) for AMF development alone. It was shown that the physical separation of AMF and Ri T-DNA transformed roots enhanced sporulation and hyphal density [130,142]. This compartmentalized system has since been widely used to study nutrient transport (e.g., phosphorus), carbon uptake, and the metabolism and transport of lipids in arbuscular mycorrhiza [143,144,145]. It has also facilitated the study of AMF hyphal architecture, sporulation, and spore ontogenesis, improving the efficiency of spore production [104,146].

Approximately 20 species of AMF have been propagated using the ROC system, but less than 10 species have been successfully maintained in vitro in international collections and are capable of growing and producing propagules after subculturing. Therefore, diversifying the transformed root species used in ROC systems is crucial for optimizing host–AMF combinations, which will support the propagation of a wide variety of mycorrhizal species and enhance germplasm collection and AMF research [112].

#### 3.2.2. Bipartite/Tripartite Culture Systems

A tripartite in vitro culture system with whole strawberry microplants was used to investigate the influence of mycorrhizal fungi on plant growth [147]. The micropropagated plantlets were inoculated with AMF-colonized transformed carrot roots, grown on M medium. Following root induction in micropropagated plantlets and the establishment of primary mycorrhizae, secondary symbiosis was achieved by removing the rooting medium from cellulose plugs through suction and rinsing the plugs with sterile distilled water under sterile conditions. After the washing procedure, M medium was aseptically added to the Sorbarod plugs supporting the plantlets. These plugs were then placed in contact with the primary mycorrhizae within the culture vessel under aseptic conditions [147]. Through CO_2_ enrichment in this tripartite culture system, the authors observed not only a high percentage of mycorrhizal establishment but also significant proliferation of the inoculum and enhanced growth of the micropropagated plantlets. This study highlighted the crucial role of carbon sources in supporting autotrophy in micropropagated plantlets and stimulating mycorrhizal colonization [147]. Similar results were obtained in a bipartite system, in which strawberry microplantlets were inoculated with unsterile spores in the presence of a carbon source [148]. This experiment showed that AMF improved the quality of microplantlets during and after acclimatization, compared to in vivo plants.

These systems were reported to have some limitations. In the tripartite culture system, three components were involved: the plant in vitro, the AM fungus, and the root organ culture. This complexity could potentially disrupt the typical one-to-one interactions between organisms. Similarly, in the bipartite culture system using polyurethane foam, it was challenging to non-destructively examine the development of the extraradical mycelium and its micro-morphological characteristics, such as colony architecture [149].

#### 3.2.3. The ACS

The ACS was developed to address the limitations of the ROC system. Since transformed roots lack photosynthetic tissues, certain physiological studies—such as examining the mechanisms of mineral and carbohydrate transport between the fungus and plant shoots—cannot be performed. Consequently, a functional whole plant is needed to study both the effect of shoots on AMF development and the plant’s systemic response to mycorrhization [149]. In the ACS, the mycorrhizal association is established between the fungal inoculum and the roots of an autotrophic plant, with the shoot extending through a pre-established hole in the Petri dish, allowing development in an aerial environment [149]. In this system, a mono-compartmental Petri dish containing MSR medium (lacking sucrose and vitamins) was used to associate potato plantlets with spores of *Glomus intraradices*, grown on excised transformed carrot roots. The shoot extended beyond a hole in the side of the Petri dish lid, while the roots remained on the surface of the medium with isolated spores. After 22 weeks of culture, the system produced more than 12,000 spores, which were capable of reinfecting new hosts in vitro and completing the fungal lifecycle. This system can be used as a continuous tool for AMF propagation [149].

#### 3.2.4. The Half-Closed AM-Plant System (HAM-p)

A modified in vitro ACS, known as the half-closed AM-plant system (HAM-p), was described [150]. Unlike the traditional ACS setup, HAM-p involves a bi-compartmental Petri dish rather than a single-compartment configuration. In this system, the root system of the host plantlet is placed on the medium surface within the first compartment, the RC, where fungal spores are co-inoculated alongside the roots. The second compartment, designated as the HC, supports the growth of only the extraradical hyphae, preventing root intrusion. The plant shoot emerges through a pre-established opening in the lid or lateral wall of the Petri dish, allowing aerial development [126].

#### 3.2.5. The Arbuscular Mycorrhizal–Plant (AM–P) In Vitro Culture System

Due to sterility issues and medium depletion, modifications of the HAM-p system were made [150]. The modified system, called the arbuscular mycorrhizal–plant (AM–P) in vitro culture system, also uses a bi-compartmental Petri plate comprising an RC and an HC. The primary distinction from the HAM-p system is the shoot environment: whereas the HAM-p design allows the plant shoot to grow in open air outside the Petri dish, the AM–P system maintains sterility by directing the shoot into a vertically attached sterile tube—referred to as the shoot compartment (SC)—affixed to the top of the Petri plate. This system demonstrated AMF capacity to accumulate, translocate, and transfer radiocaesium (Cs) to *Medicago truncatula* [126].

#### 3.2.6. The MDP System

As previously noted, the ROC system has been used for in vitro studies of AMF symbiosis and for the large-scale production of mycorrhizal spores. However, the absence of plant shoots in this system has constrained investigations into key physiological processes such as carbon transport from shoots to the fungus and the reciprocal transfer of phosphorus (P) and/or nitrogen (N) from the fungus to the shoots.

**Figure 1:**
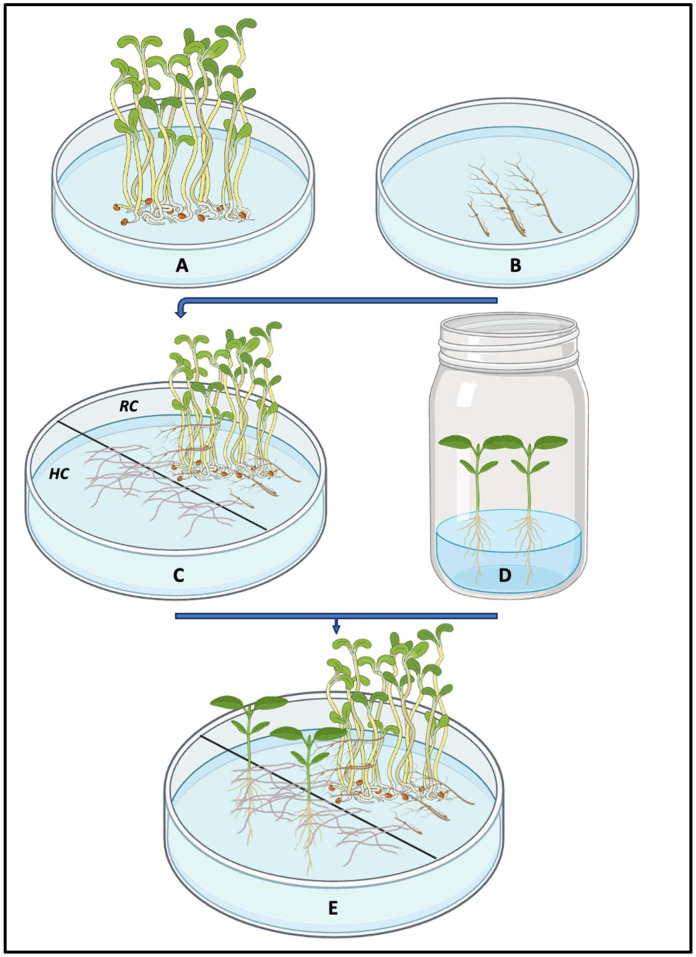
Schematic representation of the mycelium donor plant (MDP) system used for in vitro mycorrhization. (**A**) In vitro germinated seedlings of *Medicago truncatula*. (**B**) Ri T-DNA-transformed carrot roots bearing mycorrhizal spores, cultured on MSR medium without sucrose and vitamins. (**C**) Co-culture of *M. truncatula* and Ri-T DNA-transformed carrot roots bearing spores in the root compartment (RC). Extraradical mycelium with spores developed into the hyphal compartment (HC). (**D**) In vitro cultured plants prepared for in vitro mycorrhization. (**E**) In vitro mycorrhization on MSR medium without sucrose and vitamins. Adjustments can be made according to specific experimental conditions (e.g., plant species, fungal strain, culture conditions). “Created with BioRender.com”.

In recent years, whole-plant systems have been developed to overcome the limitations of root-only hosts and to address related concerns. One of the most notable innovations is the MDP system, which is based on a monoxenic, typically two-compartment setup using solid culture media. In this system, bicompartmental Petri dishes (e.g., 90 mm in diameter) physically separate the RC from the HC (Figure 1). Small openings (~2 mm in diameter) are made in both the base and lid of the plates. Each compartment is filled with solidified medium, such as MSR medium lacking sucrose and vitamins [86]. Rooted micropropagated plantlets are cultured in association with an actively growing extraradical mycelium that extends from an AMF-colonized donor plant (Figure 1). The MDP system was designed to demonstrate the ability of extraradical mycelium from an autotrophic donor to colonize in vitro plantlets and reproduce the fungal life cycle [86].

In a slightly different configuration, the system comprised two Petri dishes of different sizes—typically 55 mm (i.e., RC) and 145 mm (i.e., HC) in diameter—both filled with the same culture medium, such as MSR, lacking vitamins and sucrose [39]. A donor plant is introduced to enhance and promote the mycorrhization of a target plantlet. The smaller dish (referred to as RC) is placed within the larger one (HC). In RC, the donor plant’s roots are co-cultivated with arbuscular mycorrhizal spores. *Medicago truncatula* is commonly selected as the donor species due to its high mycotrophic nature [39]. The host plantlet is cultivated in HC, where fungal hyphae develop. The extraradical mycelium, bearing spores, extends from RC to HC and facilitates colonization of the host plantlet’s roots.

The MDP system is an adaptation of the autotrophic in vitro culture approach introduced by Voets et al. [149] and enables fast, extensive, and homogenous colonization of plant roots [86]. The MDP system has been successfully applied to some plant species (Table 1), including banana [39], rubber tree [151], potato [40], pear [41], date palm [42], and argan [43]. These studies have demonstrated that the MDP system is a powerful tool for producing homogeneous, highly colonized in vitro plantlets in a short time. It also facilitates the pre-adaptation of micropropagated plantlets to ex vitro conditions, potentially speeding up their development for field transfer.

## 4. In Vitro Mycorrhization for the Production and Commercialization of AMF, Mycorrhizal Plants and Secondary Metabolites

Mycorrhizal symbiosis provides significant economic benefits due to its positive effects on plant growth, crop yield, plant nutrition, quality, stress tolerance, pathogen protection, and soil structure improvement [152]. In vitro culture of AMF has proven to be an effective tool for large-scale production and commercialization of mycorrhizae. Many studies have used this method to mass-produce AMF, offering advantages over conventional methods by achieving a several-fold increase in spore/propagule production in less time and space [153]. The process involves extracting viable propagules from soil, surface sterilization, and optimizing growth conditions for germination under aseptic conditions. Once mass-produced, propagules are formulated in a usable form and stored for later application to the target plant. The formulation should be simple, cost-effective, and easy to transport and apply, while the fungal propagules must remain viable during storage and distribution across a wide range of temperatures [154]. Currently, commercial mycorrhizal inocula are produced using a variety of methods, including pots, nursery plots, containers with different substrates and plants, aeroponic systems, nutrient films, and in vitro cultivation [155]. Various forms of mycorrhizal inoculum are available, including tablets, pellets, granules, powdered inoculum, gel beads, and balls [156]. Formulating AMF inoculum with a carrier material such as vermiculite, peat, perlite, inorganic clay, or sand allows effective dispersion and targeted application [154]. However, if the carrier is too adhesive and does not dissolve upon watering, the effectiveness of the inoculum may be reduced, as roots and mycorrhizal propagules may not associate properly [156].

Other formulations, such as encapsulation, have also been used [157]. In this method, spores or mycelial fragments are combined with an alginate solution, and the living propagules become trapped in the gel matrix through polymerization. This gel facilitates substrate diffusion, protects the inoculum during storage and from harsh environmental conditions after field application, and can be easily supplemented with nutrients [158]. The capsule acts as a protective barrier from adverse environmental conditions and serves as a nutrient reservoir, promoting fungal growth and stimulating root development, which helps mycelium establish a symbiotic relationship with the plant roots [159]. Encapsulation also stabilizes the biological properties of mycorrhizal roots and isolated vesicles, and is essential for long-term preservation techniques such as lyophilization or cryopreservation. These methods protect the AMF inoculum from the potentially harmful effects of cryoprotectants and from mechanical and oxidative stress during storage. Other preservation techniques, such as drying and cold storage [160,161], L-drying [162], and liquid nitrogen storage [163], have also been reported.

Recent advancements in AMF inoculant technology have increasingly prioritized the enhancement of formulation stability, propagule viability, and delivery efficacy through encapsulation and the use of advanced carrier systems. Various strategies have been developed to protect AMF propagules from environmental stressors while extending shelf life and improving field performance. Encapsulation techniques using natural polymers such as chitosan, carrageenan, gelatin, laponite, and other biodegradable matrices have emerged as promising approaches to improve the physical stability, environmental resistance, cell viability, and biological efficacy of AMF inoculants [164]. These systems generate protective microenvironments that shield AMF spores from desiccation, ultraviolet radiation, temperature fluctuations, humidity, and mechanical damage during storage and transportation [164]. In parallel, formulation efforts have focused on improving scalability and cost-efficiency through advancements in substrate-based cultivation, bioreactor systems, and solid-phase media. Strain selection strategies increasingly emphasize genetic stability and ecological compatibility to ensure consistent performance across diverse agroecosystems. Additionally, the co-inoculation of AMF with other plant-beneficial microorganisms in multispecies microbial consortia is being explored to further enhance plant growth and agricultural productivity [165].

Despite these advances, a comprehensive evaluation of commercial AMF inoculants has revealed persistent challenges, including reduced propagule viability, contamination with phytopathogens, and limited colonization potential, highlighting the urgent need for standardized quality control measures and improved production methodologies [166]. The successful development of high-quality bioencapsulated AMF products depends on multiple factors, including the selection of suitable polymers, capsule size, encapsulation technique, and the optimization of chemical and physical parameters during production [164]. Therefore, the refinement of formulation technologies must be accompanied by regulatory frameworks that establish clear quality standards for AMF-based biostimulants. Such developments are essential to support the broader commercialization and integration of AMF inoculants into sustainable agricultural practices, ultimately contributing to the development of more reliable, scalable, and effective solutions for crop production.

Commercial inoculum production primarily uses AMF species from the *Glomeraceae*, along with *Gigasporaceae*, *Scutellosporaceae*, and *Acaulosporaceae* families [167]. *Glomus* species are the most widely marketed commercial species, commonly incorporated into various bioformulations [2,165]. Although in vitro-produced inoculum may be more expensive than greenhouse-propagated inoculum, it offers pure, contaminant-free, and reliable material for research and commercial applications, providing a solid foundation for inoculum production in both fundamental research and applied technologies [155]. Combining mycorrhization with the photoautotrophic micropropagation (PM) system in vitro is an effective strategy to mitigate the stress-induced damage to plantlets both in vitro and ex vitro. This approach also facilitates the mass production of high-quality mycorrhizal transplants and inoculants through various cultivation strategies [168].

The integration of mycorrhization into micropropagation systems represents a promising strategy for the large-scale production of mycorrhizal plants. This approach not only facilitates the mass multiplication of AMF inoculants and mycorrhizal plants, but also enhances the resilience of micropropagated plantlets to environmental stresses during in vitro and ex vitro phases [168]. In vitro propagation via tissue culture offers a potent tool for the large-scale multiplication and conservation of plant genetic resources, ensuring year-round availability for research, cultivation, and commercial applications. When integrated with mycorrhization techniques, this approach significantly enhances the survival, establishment, and overall vigor of micropropagated plantlets during the critical transition from in vitro conditions to ex vitro environments [119]. The combination of mycorrhization and tissue culture techniques thus provides a viable pathway for large-scale propagation and improving plant establishment and survival during acclimatization and field transfer.

Numerous case studies support the feasibility of this method across diverse plant species for the large-scale production of mycorrhizal plants. For example, it was demonstrated that in vitro mycorrhization of banana (*Musa acuminata*) with *Rhizophagus irregularis* significantly improved the acclimatization success and accelerated the development of plantlets, highlighting its potential for large-scale production of mycorrhizal plants [39]. Similarly, it was reported that the MDP system enabled efficient colonization (up to 55%) of potato (*Solanum tuberosum* (L.)) within just 12 days of contact with AMF hyphae, underscoring its practical relevance for rapid production of mycorrhizal plants, which are likely to exhibit enhanced resilience to environmental stresses [40]. Further evidence showed that in vitro mycorrhization of pear (*Pyrus communis*) with *R. irregularis* not only improved acclimatization outcomes but also enhanced developmental rates, offering a viable system for large-scale production of robust mycorrhizal pear plantlets, facilitating earlier and more successful transfer to field conditions [41].

The application of this technology has also been extended to the tree species of ecological and economic importance. In vitro co-culture of argan (*Argania spinosa* (L.) Skeels) with *R. irregularis* resulted in improved growth performance and post-transplant recovery, suggesting its suitability for both large-scale propagation and ecological restoration of the argan ecosystem [43]. Similarly, the successful mycorrhization of date palm (*Phoenix dactylifera* (L.)) in vitro with *R. irregularis* was demonstrated, which facilitated better adaptation during acclimatization and potentially enhanced performance under field conditions [42]. Collectively, these findings confirm the efficacy of in vitro mycorrhization as a powerful tool in plant biotechnology, offering significant advantages for plant propagation, AMF inoculant production, and sustainable agriculture.

Furthermore, AMF enhance the production of secondary metabolites in medicinal plants, making their association a promising technology for the commercial production of secondary metabolites with pharmacological, medical, and cosmetic applications. Along this line, AMF inoculation has been shown to increase the production of various secondary metabolites, including alkaloids, terpenoids, phenolics, and saponins [169]. In summary, combining mycorrhization with in vitro propagation offers a sustainable approach to mass-producing both high-quality plantlets and AMF inoculants. This method also facilitates the proliferation of plant species that are difficult to propagate, while promoting the enhanced production of secondary metabolites and bioactive compounds.

## 5. Effects of In Vitro Mycorrhization on Plant Morphology, Physiology, and Biochemical Compounds

Although limited in number, studies on in vitro mycorrhization have demonstrated its positive effects on stem and root growth, plant acclimatization, and subsequent growth under both normal and stressful environmental conditions [2].

The autotrophic culture system has been successfully applied to various potato cultivars [149]. In their study, the roots of potato plantlets were co-cultivated with transformed carrot roots colonized by *Glomus intraradices* (MUCL 43194), which stimulated primary root proliferation and enhanced root branching. After 6 weeks, extensive internal root colonization was observed, accompanied by significant extraradical hyphal development and the formation of spores, arbuscules, and vesicles. Moreover, stolon and microtuber formation became evident after 10 weeks, with stolons reaching heights of 15–20 cm and microtubers attaining diameters of 8–10 mm by week 22.

In another study, in vitro mycorrhization of potato (*Solanum tuberosum* L. cv. Desirée) was achieved using the MDP system, in which potato plantlets were associated with an extensive mycelial network of *Rhizoglomus intraradices* (MUCL 41833) through *Medicago truncatula* plants [40]. This system promoted the healthy potato plant growth in MSR medium as early as 9 days after contact in the HC of the MDP setup. High levels of root colonization (55%) were observed within 12 days of exposure to the fungal network. Notably, potato plants were able to regenerate fungal colonies when transferred to fresh media, producing abundant extraradical structures such as mycelium and spores. Furthermore, inoculation with *R. intraradices* extracellular mycelium from a donor plant enabled rapid and efficient colonization in vitro, significantly reducing the time required for the colonization of potato [40]. An in vitro mycorrhization system for Castilla blackberry (*Rubus glaucus*) plants in autotrophic culture systems was developed [170]. Micropropagated plants were successfully associated with *Glomus* sp. (GEV02), resulting in significantly higher production of both aerial and root biomass compared to the control. Besides, the formation of typical symbiotic structures, such as good intraradical colonization, production of arbuscules and vesicles, as well as the development of extraradical mycelium with branched hyphae and the formation of new spores, were observed [170].

Micropropagated *Hevea brasiliensis* plants, cultivated using the MDP system, were successfully colonized by the arbuscular mycorrhizal fungus *Rhizophagus irregularis* (MUCL 41833). Under elevated CO_2_ concentrations in combination with 2-morpholineoethanesulfonic acid monohydrate, a high frequency of arbuscule formation and abundant production of spores and vesicles were observed, accompanied by a significantly increased rate of root colonization. These effects were most prominent in newly formed roots, suggesting a more pronounced establishment of the symbiotic association under these experimental conditions [151].

Banana (*Musa acuminata*) plantlets were mycorrhized with *R. irregularis* (MUCL 41833) using the MDP system. After five weeks of contact with the extraradical mycelial network, 80% of the root system was colonized. At this stage, no significant differences were observed in pseudostem height, pseudostem diameter, or shoot and root dry weights between mycorrhizal and non-mycorrhizal (control) plants. However, during the acclimatization phase, these growth parameters improved notably in the mycorrhizal plants. Specifically, five and seven weeks after transfer to a peat–sand substrate, in vitro mycorrhizal banana plantlets exhibited significantly greater pseudostem height and diameter, as well as increased shoot and root biomass, compared to control plants [39].

In vitro mycorrhization of pear (*Pyrus communis*) was successfully achieved using the MDP system and an inoculum composed of *R. irregularis* (strain INCAM11) spores/mycelium and chicory root segments. This led to the formation of typical intraradical structures (i.e., hyphae, arbuscules, spores/vesicles). Additionally, the process resulted in improved acclimatization survival, enhanced growth and development, better rooting (in terms of both number and length), and altered nutrient uptake [41]. The MDP system was also used to colonize date palm (*Phoenix dactylifera* L.) plantlets with *R. irregularis* (MUCL 41833). Root colonization was achieved after 10 weeks, with the presence of hyphae, vesicles, spores, and arbuscules. While no significant differences were observed in above-ground parameters (such as the number, length, and weight of leaves) between mycorrhizal plants and controls, a greater number of secondary and tertiary roots were observed in mycorrhizal plants [42]. Argan (*Argania spinosa*) seedlings were colonized via the MDP system with *R. irregularis* (MUCL 41833). Root colonization, characterized by the presence of arbuscules, vesicles/spores, and hyphae, was observed after 8 weeks of growth in the extraradical mycelium network of the fungus. Mycorrhizal plants also showed significant growth improvements, with increased concentrations of chlorophyll a and b in the leaves. Thus, in vitro mycorrhization of argan seedlings resulted in effective root colonization and enhanced plant growth after acclimatization [43].

In vitro mycorrhization of *Dendrocalamus asper* was accomplished using a solution of *Rhizoglomus clarum* (CNPMS05) in liquid culture medium containing the plants to be mycorrhized. On MSR medium, 16.6% of the plants were colonized, with evidence of hyphae, spores, and vesicles. Mycorrhization significantly enhanced root formation, whereas it had no significant effect on stem number. When cultured on ½ MS medium, the colonization rate was 25% [44]. In potato plantlets, mycorrhization with *Glomus intraradices* enhanced the synthesis of key endogenous hormones, such as auxins, cytokinins, and gibberellins, contributing to improved growth, development, and mini-tuber production [171].

Some studies have used micropropagated plants and applied mycorrhization during the acclimatization stage. For example, Azcón-Aguilar et al. [172] showed that mycorrhization of micropropagated *Annona cherimola* Mill. plantlets at the beginning of their acclimatization phase induced beneficial changes in root system morphology, improving nutrient acquisition and plant development, and equipping the plantlets to better withstand stress. Micropropagated grape plantlets (*Vitis vinifera* L.) were inoculated with six single and one mixed strain of AMF during the acclimatization process. The mycorrhizal inoculation led to an accumulation of various biochemicals in the plant system, including chlorophyll, carotenoids, proline, phenols, and enzymes such as polyphenol oxidase and nitrate reductase. Mycorrhizal-treated plantlets exhibited improved survival rates and enhanced tolerance to stresses encountered during the weaning phase. Additionally, these plants showed better physiological and nutritional status, with increased RWC and photosynthetic efficiency. Furthermore, they accumulated higher concentrations of N, P, Mg, and Fe [173]. The integration of micropropagation and mycorrhizal inoculation has been identified as a valuable approach for promoting more sustainable horticultural production. This combined strategy enhanced the survival rates of artichoke plants during the acclimatization phase [174].

In sum, inoculation with active AMF cultures appears critical for the survival and growth of micropropagated plantlets, preventing transplant shock and stunted growth upon field transfer. Rapid AMF colonization also improves the physiological status of plants during acclimatization, promoting early recovery, vigorous growth, and higher biomass accumulation in mycorrhizal plants.

## 6. In Vitro Mycorrhization for Enhancing Resistance to Abiotic and Biotic Stresses

Mycorrhizal inoculation plays a crucial role in promoting plant growth and nutrient uptake, making it an effective strategy to mitigate both abiotic and biotic stresses in economically important plant species [2]. Crop production and sustainability are strongly influenced by stress factors such as drought, salinity, heavy metal toxicity, extreme temperatures, and pathogens [36]. Some studies have explored the impact of in vitro mycorrhization on host plants under stress conditions. As mentioned earlier, mycorrhizal fungi help improve environmental (mainly temperature and relative humidity) shock tolerance during the acclimatization phase by inducing morphological and physiological changes in micropropagated plants.

Tissue-cultured strawberry plantlets (*Fragaria* × *ananassa* Duch. cv. Kent) were colonized in vitro by *Glomus intraradices* using a tripartite in vitro culture system. After 4 weeks of co-culturing with AMF, water stress was induced using polyethylene glycol (PEG)-8000. It was revealed that mycorrhization of micropropagated strawberry plantlets influenced the PEG-induced changes in amino acid content. In particular, mycorrhizal plantlets treated with PEG exhibited a significant reduction in asparagine levels in the leaves, while asparagine concentrations increased substantially in the roots. Additionally, the concentrations of aspartic acid, serine, threonine, amino-N-butyric acid, alanine, and starch were higher in the roots of mycorrhizal plantlets compared to nonmycorrhizal ones. This highlights the distinct strategies employed by mycorrhizal and non-mycorrhizal plantlets to manage water stress and suggests that AMF play a key role in modulating nitrogen and carbon metabolism in response to water stress [175]. Co-culturing *Glomus lamellosum*, *G. intraradices*, and *G. proliferum* with Ri T-DNA transformed carrot roots has been reported to enhance tolerance to heavy metal toxicity, particularly to lead (Pb), by promoting the accumulation of phenolic compounds in the root cells [176]. These compounds formed stable complexes with toxic metals, reducing metal uptake and alleviating toxicity [176].

Mycorrhized plantlets may exhibit quicker adaptation to biotic stresses, induced by pathogens, through systemic acquired resistance (SAR) or induced systemic resistance (ISR) [39]. The MDP system was used to associate in vitro potato (*Solanum tuberosum* L.) seedlings with *Glomus* sp. (MUCL 41833) and demonstrated that this fungal strain induced systemic resistance in potato leaves against *Phytophthora infestans*, particularly during the early stages of infection. This was evidenced by a reduced leaf infection index. Besides, the pathogen did not affect the colonization of the roots by AMF. Additionally, the induction of Pathogenesis Related 1 (PR1) and PR2 genes in the leaves of mycorrhizal potato plantlets inoculated with the foliar pathogen further suggested that the systemic resistance conferred by the AMF was linked to the activation of these two PR genes [177]. Similarly, it was demonstrated that the hyphae of *R. irregularis*, established using the MDP in vitro system with potato plantlets infected by *P. infestans*, transmitted warning signals from diseased to healthy plants. This process activated defense responses in the uninfected plantlets, highlighting the direct role of hyphae in transmitting these signals and confirming their essential role in activating defense mechanisms in healthy plants [178]. Under saline conditions, it was found that lettuce plants inoculated with *G. intraradices* DAOM 197198 exhibited better growth potential, higher RWC, lower proline and ABA contents, and altered expression of stress-related genes compared to non-mycorrhizal plants, indicating that AMF-mycorrhizal plants are more tolerant to salt stress than non-mycorrhizal plants [179]. The association of *G. intraradices* with transformed chicory roots in a benzo[a]pyrene (B[a]P)-enriched medium was used to investigate the role of in vitro mycorrhization in enhancing resilience to oxidative stress [180]. A significant decline in AMF development was observed in response to elevated concentrations of B[a]P. Additionally, higher malondialdehyde and peroxidase activity in non-colonized roots were observed, while superoxide dismutase activity was higher in AM-colonized roots, indicating that AMF conferred tolerance to oxidative stress caused by B[a]P [180]. In micropropagated grape rootstocks, in vitro mycorrhization with AMF not only enhanced P uptake but also improved the absorption of K, Ca, and Mg. This improved nutrient acquisition helps mitigate salt stress by counteracting the harmful effects of elevated sodium and chloride levels, ultimately contributing to greater chlorophyll stability [181].

Furthermore, AMF play a key role in regulating plant water relations by enhancing transpiration rate, leaf water potential, stomatal conductance, chlorophyll content, photosynthetic efficiency, and CO_2_ fixation, particularly under drought stress. These physiological improvements enable mycorrhizal plants to maintain higher water content and photosynthetic activity compared to non-mycorrhizal counterparts, likely contributing to improved adaptation during acclimatization and in planta transfer [182].

Overall, these studies demonstrate the effectiveness of mycorrhizal associations in promoting plant health, enhancing tolerance to various environmental stresses, and improving plantlet acclimatization, thus boosting ex vitro survival. In vitro mycorrhization has been shown to improve both the morphological and physio-biochemical characteristics of plants while boosting their resistance to abiotic and biotic stresses. This highlights the potential of this system for the rapid production of stress-tolerant plants, with promising implications for agronomic productivity, economic value, and both food and ecological sustainability.

## 7. Conclusions

The association of in vitro culture systems with mycorrhization has been discussed as a powerful tool for studying plant-mycorrhizal interactions. These techniques provide controlled conditions that allow for detailed observation of the effects of mycorrhizae on plant morphology and physiology. The key role of AMF in enhancing plant health, survival, and restoration in native ecosystems, as well as their contribution to soil structure, was highlighted. The various benefits of AMF for their host plants, including improved tolerance to abiotic and biotic stresses, enhanced growth, modulation of ecosystem resilience, and maintenance of soil structure, were discussed.

Key factors influencing the success of in vitro mycorrhization include the isolation and cultivation techniques of mycorrhizae, the choice of growth medium, and the inoculation of the appropriate mycorrhizal fungi. Recent advances in in vitro mycorrhizal culture not only enhance our understanding of plant–fungus interactions but also support the development of biotechnologies for large-scale production and commercialization of AMF propagules and mycorrhizal plants. In vitro mycorrhization represents a promising strategy to support the rapid adaptation of micropropagated plantlets to ex vitro conditions. It enhances resilience to both abiotic and biotic stresses, improves nutrient uptake and water use efficiency under adverse conditions, and can potentially accelerate plant development for successful field establishment. Therefore, this technique is highly effective for the mass propagation of plants with increased tolerance to environmental stress. Finally, in vitro mycorrhization represents an innovative and economically viable alternative to chemical fertilization, reducing environmental pollution. Future research should focus on optimizing AMF production and conservation techniques, extending the application of this approach to a wider range of crops with high agronomic and economic value, and exploring the long-term impacts of these technologies on sustainable agriculture and ecosystem health.

## Figures and Tables

**Table 1:** Examples of reported cases of successful in vitro mycorrhization involving economically and/or ecologically important plant species.

Host Plant Species	Mycorrhizal Fungus Strain	Root System	Mycelium Donor Plant Species	Culture Medium	In vitro Mycorrhization System	Key Findings	Reference
Potato (*Solanum* *tuberosum* L). cvs. Désirée, Kennebec, Pink Fir Apple, Waycha, Rosa, and *Solanum phureja*	*Glomus intraradices* Schenck & Smith (MUCL 43194)	Transformed carrot roots (*Daucus carota* L.)	-	MSR medium lacking vitamins and sucrose	ACS system	Spore production exceeded 12,000 spores after 22 weeks of in vitro culture. Spores effectively colonized new plantlets under consistent conditions. Potato shoots reached heights of approximately 15–20 cm, with 0 to 3 microtubers (8–10 mm in diameter) formed per plant. The autotrophic culture system enables sustained in vitro culture of arbuscular mycorrhizal fungi.	[149]
Rubber tree (*Hevea brasiliensis*) genotype Prang Besar	*Rhizophagus irregularis* (Błaszk., Wubet, Renker & Buscot) C. Walker & A. Schüßler comb. nov. (MUCL 41833)	Ri T-DNA transformed carrot (*Daucus* *carota* L.) roots clone DC1	*Medicago truncatula.* Gaertn. cv. Jemalong A 17	MSR medium lacking vitamins and sucrose	MDP/Bi-compartmental system	Root colonization was enhanced by elevated CO_2_ levels and the addition of 2-morpholineoethanesulfonic acid monohydrate (MES) buffer to the culture medium. Newly developed root tissues exhibited colonization structures, including arbuscules and spores/vesicles.	[151]
Banana (*Musa acuminata*) cv. Grande Naine	*Rhizophagus irregularis* (Błaszk., Wubet, Renker, and Buscot) C. Walker and A. Schüßler comb. nov. (MUCL 41833)	Ri T-DNA transformed carrot (*Daucus carota* L.) hairy root clone DC1	*Medicago truncatula.* Gaertn. cv. Jemalong A 17	MSR medium lacking vitamins and sucrose	MDP/Bi-compartmental system	Banana plantlets exhibited substantial root colonization. Hyphal regrowth was observed within 2 days following the transfer of plantlets from the MSR + AMF treatment to fresh MSR medium. Initial spore formation was observed after 3 days. After 5 weeks of exposure to extraradical mycelium, root colonization exceeded 80%, with arbuscules comprising approximately 40% and spores/vesicles over 60%. Following 5 and 7 weeks of acclimatization, banana plantlets subjected to the MSR + AMF treatment exhibited significantly greater pseudostem height and diameter, as well as higher shoot and root dry biomass, compared to those from the Control/MSR and Control/MS treatments.	[39]
Potato (*Solanum tuberosum* L.) cv. Desirée	*Rhizoglomus intraradices* Schenck & Smith (MUCL 41833)	Ri T-DNA transformed carrot (*Daucus carota* L.) roots	*Medicago truncatula* Gaertn cv. Jemalong, strain A-17	MSR medium lacking vitamins and sucrose	MDP/Bi-compartmental system	A high level of root colonization (55%) was achieved in potato plants just 12 days after contact with the mycelial network. Production of more than 1400 spores. Upon transfer to fresh medium, potato plants successfully re-established fungal colonies, producing abundant extraradical structures, including mycelium and spores.	[40]
Pear (*Pyrus communis* L.) cv. Arbi	*Rhizophagus irregularis* (INCAM11)	Ri T-DNA transformed chicory (*Cichorium intybus*) roots	*Medicago truncatula* cv. Salsa	MSR medium lacking vitamins and sucrose	MDP/Mono-compartmental system	After 3 to 4 weeks of incubation, approximately 30% of the pear roots were colonized. Hyphae, arbuscules, spores/vesicles, and appressoria were observed in the pear roots. Within 1 week of transferring the pear plantlet roots into the mycelial network, primary roots began producing new secondary roots. Mycorrhization promoted the formation of an abundant, extensively branched root system. *Rhizophagus irregularis* colonization influenced root system architecture and nutrient profiles in acclimatized pear plantlets.	[41]
Argan (*Argania spinosa* (L.) Skeels)	*Rhizophagus irregularis* (Błaszk. Wubet, Renker & Buscot) C. Walker & A. Schüßler comb. nov. (MUCL 41833)	Ri T-DNA transformed carrot (*Daucus carota* L.) roots	*Medicago truncatula* Gaertn. cv. Jemalong A17	MSR medium lacking vitamins and sucrose	MDP/Bi-compartmental system	Root colonization, evidenced by the presence of arbuscules, hyphae, and vesicles/spores, was observed after 8 weeks of growth within the extraradical mycelial network, reaching 30–50% total colonization by week 11. Plants successfully acclimatized during the transition from in vitro to greenhouse conditions, maintaining a high level of root colonization even after five months.	[43]
Date Palm (*Phoenix dactylifera* L.) cv. Boufeggouss	*Rhizophagus irregularis* (Błaszk. Wubet, Renker & Buscot) C. Walker & Schuessler (MUCL 41833)	Ri T-DNA transformed carrot (*Daucus carota* L.) roots clone DC1	*Medicago truncatula* Gaertn. c.v. Jemalong strain A17	MSR medium lacking vitamins and sucrose	MDP/Bi-compartmental system	At 10 weeks post-inoculation, date palm roots exhibited successful colonization, along with enhanced total root length and increased formation of secondary and tertiary roots. Root tissues contained hyphae, vesicles, spores, and arbuscules.	[42]
